# Enhancing the quality of international orthopedic medical mission trips using the blue distinction criteria for knee and hip replacement centers

**DOI:** 10.1186/1471-2474-14-275

**Published:** 2013-09-23

**Authors:** Kyle E Dempsey, Roya Ghazinouri, Desiree Diez, Luis Alcantara, Carolyn Beagan, Barbara Aggouras, Monica Hoagland, Thomas S Thornhill, Jeffrey N Katz

**Affiliations:** 1 Orthopedic and Arthritis Center for Outcomes Research, Brigham and Women’s Hospital, 75 Francis St, Boston, MA 02115, USA; 2 Department of Orthopedic Surgery, Brigham and Women’s Hospital, 75 Francis St, Boston, MA 02115, USA; 3 Department of Rehabilitation Services, Physical Therapy, Brigham and Women’s Hospital, 75 Francis Street, Boston, MA 02115, USA; 4 Department of Orthopedic Surgery, Hospital General de la Plaza de la Salud, Santo Domingo, Dominican Republic; 5 Department of Anesthesiology, Perioperative, and Pain Medicine, Brigham and Women’s Hospital, 75 Francis Street, Boston, MA 02115, USA; 6 Harvard School of Public Health, 677 Huntington Ave, Boston, MA 02115, USA; 7 Division of Rheumatology, Immunology, and Allergy, Brigham and Women’s Hospital, 75 Francis Street, Boston, MA 02115, USA

**Keywords:** Total joint replacement, International medical mission, Dominican, Arthroplasty, Program evaluation

## Abstract

**Background:**

Several organizations seek to address the growing burden of arthritis in developing countries by providing total joint replacements (TJR) to patients with advanced arthritis who otherwise would not have access to these procedures. Because these mission trips operate in resource poor environments, some of the features typically associated with high quality care may be difficult to implement. In the U.S., many hospitals that perform TJRs use the Blue Cross/Shield’s Blue Distinction criteria as benchmarks of high quality care. Although these criteria were designed for use in the U.S., we applied them to Operation Walk (Op-Walk) Boston’s medical mission trip to the Dominican Republic. Evaluating the program using these criteria illustrated that the program provides high quality care and, more importantly, helped the program to find areas of improvement.

**Methods:**

We used the Blue Distinction criteria to determine if Op-Walk Boston achieves Blue Distinction. Each criterion was grouped according to the four categories included in the Blue Distinction criteria— “general and administrative”, “structure”, “process”, or “outcomes and volume”. Full points were given for criteria that the program replicates entirely and zero points were given for criteria that are not replicated entirely. Of the non-replicated criteria, Op-Walk Boston’s clinical and administrative teams were asked if they compensate for failure to meet the criterion, and they were also asked to identify barriers that prevent them from meeting the criterion.

**Results:**

Out of 100 possible points, the program received 71, exceeding the 60-point threshold needed to qualify as a Blue Distinction center. The program met five out of eight “required” criteria and 11 out of 19 “informational” criteria. It scored 14/27 in the “general” category, 30/36 in the “structure” category, 17/20 in the “process” category, and 10/17 in the “outcomes and volume” category.

**Conclusion:**

Op-Walk Boston qualified for Blue Distinction. Our analysis highlights areas of programmatic improvement and identifies targets for future quality improvement initiatives. Additionally, we note that many criteria can only be met by hospitals operating in the U.S. Future work should therefore focus on creating criteria that are applicable to TJR mission trips in the context of developing countries.

## Background

Improved longevity in developing countries has led to the rise of chronic diseases, including arthritis [1,2]. In developed countries, total joint replacement (TJR) is often used to address symptomatic advanced arthritis. Total hip and knee replacements have been shown to enhance quality of life by improving function [3] and decreasing pain [4]. Although TJRs are cost effective in developed countries [5,6], the high cost of these procedures have made financing them difficult in developing countries. Despite the high costs of most surgical interventions, the WHO and other health organizations have called for renewed focus on building developing countries’ surgical capacities [7,8]. To meet these needs, organizations such as Operation Walk (Op-Walk) Boston have annual mission trips to provide pro-bono total knee and total hip replacements to people from developing countries while concurrently building surgical capacity by educating local physicians and surgical teams about TJR [9].

Organizations that provide surgical care abroad seek to provide the highest quality care possible, although the effectiveness of these short-term medical mission programs has recently been debated [10]. Ideally, all medical mission trips would offer services with quality that is equal to the services offered in developed countries. Although some attempts have been made to standardize surgical procedures during medical mission trips [11,12], quality criteria have not been established for TJR medical mission trips. One set of guidelines commonly used as a benchmark of TJR quality in the U.S. is the Blue Cross/Shield’s “Blue Distinction Center for Knee and Hip Replacement” criteria, which provide quality benchmarks for a range of program features including structural elements, processes, provider certifications, and reporting. Although the Blue Distinction criteria were developed to assess TJR program quality in developed countries, these criteria might also help medical mission groups to evaluate and improve the care they provide in developing countries.

This report provides the first attempt to evaluate the quality of care offered by medical missions based on TJR quality standards from developed countries. We also demonstrate that this kind of evaluation can help medical mission organizations identify areas of programmatic improvement.

## Methods

### Setting

The Dominican Republic is a small country (population 10,056,000) in the Caribbean Sea that shares the island of Espanola with Haiti. As of 2011, the country’s per capita income was approximately $9,300 [13]. Citizens have access to a free state health care plan, which provides basic primary care coverage, and private clinics provide most specialty care. The nation’s capital, Santo Domingo, is home to several private hospitals, including the Hospital General de la Plaza de la Salud. In 2008, when the Op-Walk Boston team made their first trip to the D.R., the hospital performed fewer than 20 TJRs annually, though this number has grown to more than 100 cases annually. Other private hospitals throughout the country also provide hip and knee replacements to patients who are able to pay for the procedures’ high costs, but information regarding the number of joint replacements and the outcomes of these other joint replacement programs is not available.

### Operation walk Boston team

Op-Walk Boston is part of the national Operation Walk organization [14]. The Boston team has made annual service trips to the D.R. since 2008. The team consists of approximately 50 individuals, including orthopedic surgeons, anesthesiologists, internists, physical therapists, physician assistants, surgical and medical nurses, operating room personnel, medical students, and other staff. The Op-Walk Boston team works closely with their Dominican colleagues at La Hospital General de La Plaza de La Salud in Santo Domingo to identify low-income Dominican patients with severe joint disease; the team provides pro-bono knee and hip replacements for these patients during its annual trips.

### Data collection and analysis

Using the Blue Cross/ Shield’s selection criteria for Blue Distinction Centers for Knee and Hip Replacements, we determined if Op-Walk Boston’s joint replacement program meets the Blue Distinction criteria (scores at least 60 out of 100 possible points) [15]. The study’s main author compiled the Blue Cross/Shield’s criteria and independently reviewed each criterion with two of Op-Walk Boston’s program directors during in-person interviews. The directors independently responded to each criterion with “meet”, “do not meet”, or “unsure”. For criteria that produced conflicting results, the criteria were sent to an independent person familiar with the specific criteria in question and this third party’s response served as a tie-breaker. Similarly, for all criteria that lacked responses from both directors, the study’s main author redirected the question to an independent team member who had the most knowledge of that criterion.

Full points were awarded for criteria that the program replicates exactly and zero points were given for criteria that are not replicated. If a criterion was not applicable outside of the United States, zero points were awarded and the criterion was labeled “not applicable”. For all criteria that are not replicated, we interviewed a leader from each clinical and administrative team to see if they compensate for failing to meet the criterion by introducing an alternative strategy or process to enhance quality. We also interviewed these key informants to see if there are barriers that prevent them from adopting certain criteria.

We classified the criteria as “General/Administrative”, “Process”, “Structure”, and “Outcomes and Volume” categories to follow widely-used frameworks for quality assessment [16] and to match the subcategories established in the Blue Distinction criteria. The scores from each category were graphed to represent visually the percentages of points that were met, not met, or somehow compensated for in each category. Points marked as “informational” represent criteria that are not formally scored in the Blue Cross/Shield’s criteria but are still important when evaluating joint replacement programs. Points marked as “required” are deemed essential for achieving the Blue Cross/Shield’s distinction.

## Results

### General criteria for all blue distinction centers

In the general criteria section, the program received 14 out of a possible 27 points (Table 1). The program lost four points because it lacks formal conflict of interest policies and it lost seven points because it does not collaborate with several U.S.-based quality improvement organizations. Two points were lost because it does not participate in the Surgical Care Improvement Project, although the program does compensate by following best practice surgical care guidelines.

**Table 1:** Blue distinction criteria, points awarded, accommodations made to meet the criteria, and barriers to criteria’s implementation for general and structure criteria

**Criteria**	**Points earned out of total**	**Explanation**	**Accommodation**	**Barrier**
**General criteria for all blue distinction centers**				
Facility must be an inpatient acute care hospital that provides comprehensive inpatient care (e.g., Emergency Room, Intensive Care and other specified services)	Required	Criterion met.	N/A	N/A
Full facility accreditation by a CMS-deemed national accreditation organization	Required	Criterion not met.	Hospital is working to meet the Joint Commission’s accreditation criteria.	N/A
Facility participation in IHI with a commitment to patient safety, including formal commitment to at least 6 improvement campaigns (i.e., initiatives)	0/2	Criterion not applicable because IHI does not work in the Caribbean.	Program has engaged in quality improvement measures from IHI’s list of QI initiatives	IHI does not currently operate in the DR.
Facility publicly reports on the Leapfrog Web site via the Leapfrog Group Quality and Safety Hospital Survey	0/1	Criterion not applicable because Leapfrog does not work in D.R.	N/A	The Leapfrog Group does not evaluate international hospitals.
If facility does not report to Leapfrog, facility participates in other initiatives that encourage the sharing of best practices, incorporates data feedback for objective analysis, and promotes collaborative improvement	Optional	Criterion met.	N/A	N/A
Alternate initiatives will be reviewed on a case-by-case basis				
Facility accepts the Association of American Medical Colleges (AAMC) principles for all clinical trials	1/1	Criterion met. Hospital participates in three multicenter trials, follows AAMC principles.	N/A	N/A
Facility uses a certified electronic medical record (EMR) certified by the Certification Commission for Healthcare Information Technology (CCHIT)	0/1	Criterion not met.	The hospital uses the LOLCLI 9000 EHR by LOLIMSA.	N/A
Facility uses an e-prescribing program to facilitate communication that meets the standards set forth in the 2003 Medicare Modernization Act (MMA)	0/1	Criterion not applicable.	Physicians e-prescribe using an electronic medical order sent directly to the hospital’s pharmacy. Prescriptions for outpatients must be made manually.	Medicare Modernization Act’s specifications relate to specific formularies that are not relevant in D.R.
Facility has a formal process of medication reconciliation that includes:	1/1	Criterion met.	N/A	N/A
--Verification				
--Clarification				
--Reconciliation				
Facility is currently active in one of the following quality nursing excellence initiatives:	0/1	Criterion not applicable.	Hospital currently improving nursing quality, including evaluation of nurse performance, patient quality and safety education, and CME meetings	Magnet Award from ANCC requires compliance with U.S. Department of Labor and the Department of Health and Human Services (not applicable in the DR).
--Has earned the Magnet Recognition Award of the American Nurses Credentialing Center				
--Reports to the American Nurses Association’s National Database of Nursing Quality Indicators (NDNQI)				
Facility participates in HCAHPS survey and makes data publicly available on the Hospital Compare Web site for the most recent public reporting date	0/1	Criterion not applicable.	Op-Walk Boston’s research team collects data on satisfaction using surveys for patient satisfaction, and it uses this information to improve patient care.	HCAHPS is specific to U.S. hospitals.
Facility utilizes one of the following national quality improvement initiatives focused on surgical safety:	1/1	Criterion met. Op-Walk Boston uses WHO Surgical Safety Checklists.	N/A	N/A
--Universal Protocol for Preventing Wrong Site, Wrong Procedure, Wrong Person Surgery				
--World Health Organization Surgical Safety Checklist				
Facility participates in the Surgical Care Improvement Project (SCIP)	0/2	Criterion not met.	Hospital follows best practice guidelines but does not specifically follow SCIP. Working toward Joint Commission International (JCI) accreditation.	N/A
SCIP INF 1a: Prophylactic antibiotic received within one hour prior to surgical incision	1/1	Criterion met.	N/A	N/A
SCIP INF 2a: Prophylactic antibiotic selection for surgical patients	1/1	Criterion met. Medications and allergies are reviewed before selecting a prophylactic antibiotic.	N/A	N/A
SCIP INF 5: Postoperative wound infection diagnosed during index hospitalization (OUTCOME – facility tracks & internally reports data)	1/1	Criterion met.	N/A	N/A
SCIP VTE 1: Surgery patients with recommended venous thromboembolism prophylaxis ordered	1/1	Criterion met.	N/A	N/A
SCIP VTE 2: Surgery patients who received appropriate venous thromboembolism prophylaxis within 24 hours prior to surgery to 24 hours after surgery	1/1	Criterion met.	N/A	N/A
SCIP VTE 3: Intra- or postoperative pulmonary embolism (PE) diagnosed during index hospitalization and within 30 days of surgery (OUTCOME – facility tracks & internally reports data)	1/1	Criterion met.	N/A	N/A
SCIP VTE 4: Intra- or postoperative deep vein thrombosis (DVT) diagnosed during index hospitalization and within 30 days of surgery (OUTCOME – facility tracks & internally reports data)	1/1	Criterion met.	N/A	N/A
Facility’s SCIP database is able to produce procedure-specific performance reports	Informational	Criterion not met.	Op-Walk Boston’s research team evaluates each trip’s outcomes.	N/A
Facility has a policy on physician/surgeon conflict of interest	0/1	Criterion not met.	N/A	N/A
Facility publicly reports physician/surgeon conflict of interest related to financial relationships with pharmaceutical companies or device manufacturers	0/1	Criterion not met.	N/A	N/A
Facility discloses to patients prior to surgery exclusive relationships the facility has with device manufacturers or pharmaceutical companies	0/1	Criterion not met.	N/A	N/A
Facility has a written policy or process for selecting devices in the device formulary	0/1	Criterion not met.	N/A	N/A
Facility’s policy includes a mechanism for tracking FDA-recalled prosthesis and notifying patients who have received them	Informational	Criterion not met.	N/A	N/A
Facility reports incidences of device malfunction to the device manufacturer	Informational	Criterion met.	N/A	N/A
Facility has protocols for acute pain management in peri-operative surgical patients	1/1	Criterion met.	N/A	N/A
Pain management protocols are based on national guidelines:	1/1	Criterion met. Pain management protocols modeled after protocols followed in Boston-area teaching hospitals.	N/A.	N/A
--American Society of Anesthesiologists’ Practice Guidelines for Acute Pain Management in the Peri-operative Setting				
--Pain Management Standards of the facility’s accrediting agency (identified in question #8)				
Facility has an interdisciplinary workgroup/committee/team in place for implementing pain management protocols and monitoring their effectiveness	2/2	Criterion met. Team of anesthesiologists, internists, nurses, PT’s, and orthopedists reviews pain mgmt. needs.	N/A	N/A
**Structure**				
Program is currently and has been actively performing knee and hip replacement surgery since July 1, 2009 or for at least the immediately previous 12 uninterrupted months	Required	Criterion met.	N/A	N/A
Program has a formal CQI program in place for knee and hip replacement services with the following components:	2/2	All subcategories of this criterion are met.	N/A	N/A
--Collection of quality indicator data				
--Analysis of collected data				
--Identification of issues				
--Development of improvement goals				
--implementation of changes				
--Demonstration that the implemented changes improve the quality of clinical care that patients receive				
--Ongoing requirements for physician/surgeon learning and improvement and/or regularly scheduled educational conferences				
Program maintains an internal registry or database to track knee and hip replacement patients’ treatment and outcome data	5/5	Criterion met. Research team tracks outcomes with standardized surveys.	N/A	N/A
Program has a process in place to track complications in the context of a program-wide quality improvement process	2/2	Criterion met. Complications reviewed at the end of each trip and corrective actions are taken to minimize future complications.	N/A	N/A
Program has a process in place to track primary knee and hip replacement patients who return to the facility for revision of their primary procedure	1/1	Criterion met. Op-Walk Boston’s colleagues in the D.R. monitoring patients’ ongoing needs (including revision).	N/A	N/A
Program obtains and evaluates patient satisfaction specific to knee and hip replacement services with results reported back to program staff	Informational	Criterion met.	N/A	N/A
Program has a protocol in place to contact patients (or primary physicians) for follow-up and status information post-discharge	0/1	Criterion not met.	The hospital lacks a protocol for contacting patients. Follow-up consultations are scheduled by the individual doctors.	Able to contact patients, but communicating with PCPs is challenging.
Program reports to a multi-center registry or database that tracks knee and hip replacement surgery	Informational	Criterion met. Op-Walk Boston keeps a database that is shared between HGPS and the Brigham and Women's Hospital.	N/A	N/A
Program reports to at least one of the following registries or database:	0/2	Criterion not applicable.	Op-Walk Boston’s research team tracks surgical quality.	Organizations are primarily focused on US hospitals. Require expensive membership fees or purchasing other goods.
--National Surgical Quality Improvement Program (NSQIP)				
--University HealthSystem Consortium (UHC)				
--Premier Clinical Advisor				
Program plans to participate in a comprehensive national knee and hip replacement registry once one is developed	Informational	Criterion met.	Op-Walk Boston uses a database to track all knee and hip replacement outcomes.	No TJR registries exist in the DR and there are no ongoing plans to establish one.
Facility has an inpatient unit dedicated to the care of orthopedic patients	2/2	Criterion met. During the mission trip, Op-Walk Boston has an entire hospital ward dedicated exclusively to its patients and team members.	N/A	N/A
Program utilizes multi-disciplinary clinical pathways/protocols for the care of knee and hip replacement patients that include the following features:	4/4	Criteria met for all subcategories.	N/A	N/A
--Treatment goals				
--Sequence and timing of interventions				
--Active participation of a multi-disciplinary team				
--Daily milestones				
--Coordination of discharge, patient education and other patient needs				
Multi-disciplinary pathways/protocols address the full continuum of care across inpatient and outpatient settings	1/1	Criterion met.	N/A	N/A
Multi-disciplinary pathways/protocols generate standardized pre- and post- operative order sets	1/1	Criterion met. Clinical teams follow pre- and post-operative standardized work flows.	N/A	Electronic medical systems within the host hospital do not allow for automated, electronic order sets.
Program has standing orders that are utilized for the care of knee and hip replacement patients	1/1	Criterion met.	Each procedure has defined protocols. These procedures are documented in the patients’ chart.	N/A
Pathways/protocols or standing orders are placed in the medical record for daily use by all care providers	1/1	Criterion met.	N/A	N/A
Specific physician orders are required to deviate from the pathways/protocols or standing order set	1/1	Criterion met. Deviations discussed in the context of an interdisciplinary team.	N/A	N/A
Program consults resources to develop facility’s pathways/protocols or standing orders (e.g., clinical guidelines, national standards)	Informational	Criterion met. Op-Walk Boston strives to replicate the TJR process followed by the MGH and Brigham and Women's Hospital	N/A	N/A
In addition to orthopedic surgery and/or neurosurgery, other dedicated members of the multi-disciplinary care team for knee and hip replacement include:	5/8	Most criteria met. Op-Walk Boston lacks psychiatrists and psychologists, pain management specialists, and dedicated case managers.	Anesthesia team has experience in pain management, so they function as pain management specialists.	Case managers would require additional resources.
x Anesthesiology				
x Psychiatry/Psychology				
x Pain Management Specialist				
x Clinician focused on peri-operative medical management				
x Nursing				
x Physical Therapy/Occupational Therapy (PT/OT)				
x Physiatrist/Physical Medicine and Rehabilitation				
x Dedicated case managers as care coordinators for complex patients				
Program identifies departments that have at least one identified clinician who provides as-needed consultation to the knee and hip replacement team:	Informational	Criterion met.	N/A	N/A
x Cardiology				
x Endocrinology				
x Pulmonology				
x Nutrition				
x Social Services				
Program has pain management specialist(s) with subspecialty certification in Pain Medicine	Informational	Criterion not met.	Op-Walk Boston’s anesthesiologists provide all needed pain care.	N/A
Program identifies subspecialty certification(s) held by nurses on the care team:	1/1	Criterion met, although not all nurses have one of these certifications.	N/A	N/A
x Surgical nursing				
x Orthopedic nursing				
x Rehabilitation nursing				
Physical therapists on the care team maintain the American Physical Therapy Association (APTA) certification in orthopedic care	1/1	Criterion met.	N/A	N/A
Knee and hip replacement team holds multi-disciplinary team meetings or case management conferences at least monthly	1/1	Criterion met.	N/A	N/A
Surgeons performing knee and hip replacement surgery are certified or eligible for certification by the American Board of Medical Specialties, the Royal College of Physicians and Surgeons Board, or the American Osteopathic Board of Orthopedic Surgery	Required	Requirement met.	N/A	N/A
50% of knee and hip replacement surgeons have ACGME fellowship training in Adult Reconstructive Orthopedics	1/1	All surgeons are fellowship trained in Reconstructive Orthopedics.	N/A	N/A
Surgeon participation in American Board of Medical Specialties (ABMS) Maintenance of Certification (MOC)	Informational	Criterion met.	N/A	N/A

There are two “required” and three “informational” general criteria; the Op-Walk Boston program meets one of the required criteria and one of the informational criteria. The program did not meet one of the required criteria because the host hospital is not accredited by a CMS-deemed national accreditation organization. The two unmet informational criteria relate to using a Surgical Care Improvement Project (SCIP) database to produce procedure specific performance reports and to tracking FDA-recalled prostheses and contacting patients with these prostheses. The hospital does not have a SCIP database and it also has difficulty tracking patients, which makes it difficult to contact patients whose prosthesis are recalled.

### Structure

In the structure category, the program was awarded 30 out of 36 possible points (Table 1). The only criterion that the program did not fully meet involved reporting to surgical quality improvement registries and databases. The program also lost three points because it lacks three out of eight required multi-disciplinary team members: psychiatrists and psychologists, pain management specialists, and dedicated case managers. Despite lacking psychiatrists and psychologists and formally trained pain management specialists, the program compensates by having doctors and nurses work directly with patients to address their mental health needs and by having well-trained anesthesiologists who commonly provide pain management services.

Aside from the scorable criteria, there were also two criteria that were listed as “required” and seven listed as “informational”. The program met both of the required criteria and six of the informational criteria. It did not meet one informational criterion because it lacks pain management specialists who have subspecialty certification in pain management.

### Process

The program received 17 out of 20 possible points in the process category (Table 2). The program lost one point because it lacks standardized practices for case management and discharge planning. It lost an additional point because the program does not monitor care transitions for patients who are discharged to another care setting, although it does compensate by having an electronic medical record system that helps to track patients over time. It also lost a point because it lacks formal protocols that ensure patients’ op-notes and discharge summaries are made available to their PCPs upon discharge.

**Table 2:** Blue distinction criteria, points awarded, accommodations made to meet the criteria, and barriers to criteria’s implementation for process and outcomes and volume criteria

**Criteria**	**Points earned out of total**	**Explanation**	**Accommodation**	**Barrier**
**Process**				
Structured functional assessments that are routinely performed and tracked for all knee and hip replacement patients include:	3/3	Criterion met.	N/A	N/A
--Pre-operative functional assessments				
--Functional assessments four or more weeks post-operatively				
Program identifies routine pre- and post-op assessment of functional status that are used for standardized indexes (e.g., Knee Society Score or Harris Hip Score, Western Ontario and McMaster Osteoarthritis Index, SF-36, EuroQol 5-D)	Informational	Criterion met.	N/A	N/A
Program has written patient selection criteria that are applied to all adult patients referred for knee or hip replacement	1/1	Criterion met.	N/A	N/A
Patient selection criteria are developed by a multi-disciplinary team of physicians and staff	1/1	Criterion met.	N/A	N/A
Program screens knee and hip patients pre-operatively for the presence of anxiety or depression	1/1	Criterion met.	N/A	N/A
Program uses formal measures to screen pre-operatively for anxiety or depression:	1/1	Criterion met.	N/A	N/A
--Beck Depression Inventory (BDI)		Op-Walk Boston uses the mental health subscale of the SF-36.		
--The Hospital Anxiety and Depression Scale (HADS)				
--The nine-item depression scale of the Patient Health Questionnaire (PHQ-9)				
--The mental health subscale of the Health status Questionnaire Short Form-36 (SF-36)				
--Euro Qol 5-D				
Program employs or is willing to implement SDM processes with patients considering knee or hip replacement surgery	Informational	Criterion not met.	N/A	Dominican patients are accustomed to agreeing with Doctors’ recommendations.
Program provides standardized pre-operative patient education	1/1	Criterion met.	N/A	N/A
Pre-operative patient education activities include:	2/2	Criterion met. Educational sessions, classes, and print material provided.	Hospital’s staff offers reading help for all print material.	N/A
Educational group session or class				
Interactive electronic media program				
Materials provided to the patient (print, video)				
Written questionnaire completed by the patient				
Percentage of patients participating in pre-operative patient education process greater than or equal to 90%	1/1	Criterion met.	N/A	N/A
Protocol informing patients with relevant comorbidities (e.g., BMI > 40 kg/m^2^, diabetes mellitus) of the increased risks associated with knee and hip replacement surgery	1/1	Criterion met.	N/A	N/A
Program utilizes established practice standards/recommendations for the peri-operative care of knee and hip replacement patients:	2/2	Criteria met. ASA, ACC, and ADA requirements met.	N/A	Following AHA guidelines requires prolonged and repeated contact with patients.
--American Society of Anesthesiologists (ASA) Practice Advisory for Pre-anesthesia Evaluation				
--American College of Cardiology/American Heart Association (ACC/AHA) Guideline for the Perioperative Cardiovascular Evaluation for Non-cardiac Surgery				
--American Diabetes Association (ADA) Standards of Diabetes Care in the Hospital				
--AHA recommendations for Smoking Cessation - Making Hospital-Wide System Level Changes That Succeed				
Program has a thromboprophylaxis protocol in place that is specific for knee and hip replacement patients and incorporates the American Academy of Orthopedic Surgeons (AAOS) Clinical Guideline on the Prevention of Symptomatic Pulmonary Embolism in Patients Undergoing Total Hip or Total Knee Arthroplasty [THA or TKA]	1/1	Criterion met.	N/A	N/A
Program implements the following anesthesia practices:	1/1	Criterion met.	N/A	N/A
--Knee and hip replacement patients are routinely evaluated for the use of regional anesthesia				
--The program has a protocol in place for monitoring and maintaining intraoperative normothermia for appropriate knee and hip replacement patients				
Program has protocols for the assessment and treatment of physical therapy needs in the post-operative knee and hip replacement surgery patients	1/1	Criterion met.	N/A	N/A
Program identifies aspects of PT/OT care that are provided routinely (e.g., pre-operative and post-operative education, home assessment, functional	Informational	Criterion met.	N/A	N/A
assessment, readiness-for-discharge assessment)				
Standard practices for case management and discharge planning for knee and hip replacement patients include:	0/1	Criteria not met. Does not evaluate discharge needs before admission and lacks protocols for emergency evaluations and treatment post discharge.	N/A	N/A
--Evaluation for discharge needs occurs prior to the hospital admission				
--Written criteria for hospital discharge and readmission				
--Coordination of post-discharge needs (e.g., physical therapy, home care services)				
--Written protocol for emergency evaluation and treatment post discharge				
Percentage of patients admitted from home who return to home	Informational	100% return home.	N/A	N/A
Program monitors transitions of care for patients discharged to another setting (e.g., home, rehab facility) using a formal method	0/1	Criterion not met.	Patient stored in hospital’s EHR, so patient information could be tracked.	Formal tracking protocol not followed.
Program has an established protocol ensuring the operation note and discharge summary of each patient are made available to the primary care physician upon discharge	0/1	Criterion not met.	Op-Walk Boston’s colleagues follow-up with their patients for any needed post-op care.	Most patients lack PCPs and there is no care coordination infrastructure.
Program tracks receipt of the operation note and discharge summary by primary care physician	Informational	Criterion not met.	N/A	Most patients lack PCPs and there is no care coordination infrastructure.
Program utilizes services of the local Blue Cross Blue Shield case management care team to coordinate transitions of care	Informational	Criterion not met.	N/A	Blue Cross/ Shield does not operate in the DR.
**OUTCOMES AND VOLUME**				
Average and median surgeon volumes (across all surgeons actively performing TKA or THA) are at least 50 primary or revision TKA or THA procedures during reported 12 month period. Surgeons may include cases done at any facility.	Required	Criteria met.	N/A	N/A
Facility performs at least 100 total knee and total hip replacement surgeries (primary and revisions) during reported 12 month period, with at least 25 each of total knee and total hip replacements	Required	Requirement met.	N/A	N/A
Facility volume > = 250 surgeries during reported 12 month period	0/3	Criterion not met.	N/A	N/A
Facility volume > = 500 surgeries during reported 12 month period	0/2	Criterion not met.	N/A	N/A
Facility performs 50 net revisions for Total Knee and Total Hip Arthroplasty	0/2	Criterion not applicable.	N/A	N/A
(Net Volume = total reported TKA/THA revisions minus revisions performed				
< 6 months following a primary procedure where both procedures were done at the facility)				
Average LOS for primary Total Knee Arthroplasty (TKA) less than or equal to 3.5 days	3/3	Criterion met.	N/A	N/A
Average LOS for primary Total Hip Arthroplasty (THA) less than or equal to 4.0 days	3/3	Criterion met.	N/A	N/A
Average LOS for Revision Hip Replacement, Hip Resurfacing and Revision Knee Replacement	Informational	Revisions rare. Inadequate data to access criterion.	N/A	N/A
Average 30-day readmission rate for primary Total Knee Arthroplasty (TKA) less than or equal to 10%	2/2	Criterion met.	N/A	N/A
Average 30-day readmission rate for primary Total Hip Arthroplasty (THA) less than or equal to 10%	2/2	Criterion met.	N/A	N/A
Program tracks the selection, administration and discontinuation of prophylactic antibiotics for total knee replacement patients: SCIP INF 1e, INF 2e, and INF 3e	Informational	Criterion not met.	Each surgeon tracks their patients, but no programmatic level tracking.	N/A

There are six informational criteria in the process category, and the program met three of them. One unmet criterion involved using shared decision making processes with patients. Another unmet criterion involved tracking op-notes and discharge forms to ensure the patient’s PCP receives the documents. The final unmet informational criterion involved using the Blue Cross/ Shield’s case management team to track transitions of care.

### Outcomes and volume

In the outcomes and volume category, the program received 10 out of 17 possible points (Table 2). The program lost three points because the host facility does fewer than 250 TJRs annually, and it lost an additional two points because the host facility does fewer than 500 TJRs annually. It lost two additional points because the host hospital does fewer than 50 TJR revisions annually.

There are two required and two informational criteria in the outcomes and volume category. The program met both of the required criteria and did not meet either of the informational criteria. One informational criterion requests that the program publically report average lengths of stay for patients who need hip and knee revisions and the other criterion involves tracking selection, administration, and discontinuation of prophylactic antibiotics for knee replacements.

### Overall evaluation

**Figure 1:**
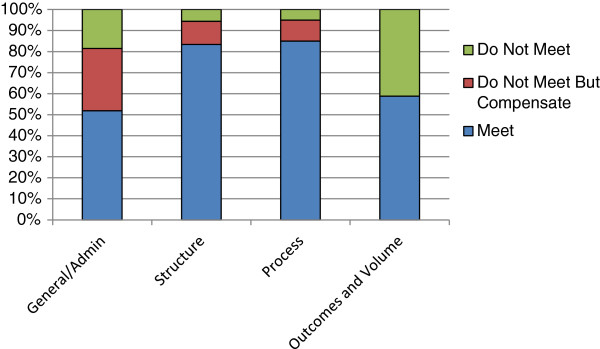
Weighted score breakdowns for criteria that are met or not met in each of the four quality categories.

The Op-Walk Boston program met enough criteria to score 71 of 100 possible points, exceeding the 60-point threshold needed to qualify as a Blue Center of Distinction (Figure 1). The program met five of the possible eight “required” criteria and 11 out of the possible 19 “informational” criteria.

Common barriers were identified that prevented some criteria from being met. First, many of the criteria require registration and participation in organizations that only exist in the United States or other developed countries. Second, many criteria were not met because they require frequent patient contact and PCP follow up. Third, some criteria require outcomes and patient tracking through online databases that are not currently available in many hospitals in the D.R.

## Discussion

In this study, we evaluated the quality of the Op-Walk Boston’s TJR medical mission trip using the Blue Cross/ Shield’s Blue Distinction Criteria for Knee and Hip Replacement Centers. This analysis showed that the program scored 14/27 in the general/administrative scoring category, 30/36 in the structure category, 17/20 in the process category, and 10/17 in the outcomes and volume category. (Figure 1) Overall, the program scored 71/100, which exceeds the 60 point threshold for Blue Distinction designation. We spoke with the program’s respective clinical teams about accommodations they make to compensate for unmet criteria. If the program did not meet a criterion and does not compensate in some way, we inquired about the barriers that prevent the program from meeting the criteria.

Although there is a growing body of quality assessment literature for medical mission trips in other fields [12], our study is the first to evaluate programmatic quality in the context of an international TJR medical mission trip. Coupled with previously published outcomes data from the Op-Walk Boston program that showed patients’ pain and function greatly improved after their TJR surgeries [17], it seems that the Op-Walk Boston team provides high quality care to the patients it serves in the D.R. Similar international joint replacement programs should note that having a dedicated research team that monitors outcomes can provide data to improve clinical care and is therefore essential in ensuring continued quality improvement in short-term international medical missions [18]. Our research team’s operation accounted for eight out of the 71 points the program received, and it also helped to meet three of the informational criteria.

In addition to demonstrating quality, the process of evaluating the Op-Walk Boston program based on the Blue Distinction criteria helped generate ideas for quality improvement initiatives. For example, the program lost a point because it does not use patient navigators. Since Op-Walk Boston’s patients often have complex social needs and may have difficulties navigating the health care system, initiating a patient navigation program may help with the hospital experience and subsequent recovery. The program also lost a point because it does not evaluate patient’s discharge needs prior to their hospital admission. This is another area we have now identified for improvement. We plan to discuss with Op-Walk’s medical, nursing and rehabilitation colleagues in the Dominican Republic culturally acceptable ways of amplifying the patient's voice in decision making. Some of the barriers we identify could be overcome with greater funding. We have discussed our findings with administrative and clinical leaders in the Dominican Republic so that the observed deficiencies can be considered for funding in subsequent budgeting processes.

When weighted scoring breakdowns were graphed according to the Blue Cross/Shield’s four major quality categories—general/administrative, structure, process, and outcomes and volume—the program’s general/administrative and outcomes and volume categories emerged as the weakest. (Figure 1) These categories contained unique obstacles that are difficult or impossible for the program to overcome. In the general/administrative category, for example, the program lost five out of a potential 27 points because the criteria requested that the program report to agencies that are primarily based in the U.S. and do not currently operate in the D.R.; these criteria are therefore not applicable to Op-Walk Boston’s TJR program.

In the outcomes and volume category, the program lost 7 points mainly because its host hospital does fewer than 250 TJRs and 50 revisions procedures annually. When Op-Walk Boston first began working in the D.R., its host hospital performed fewer than 20 TJRs annually; volume has expanded to over 100 annually. Although it does not meet the 250 annual TJR threshold requested by the Blue Cross/Shield criteria, the host hospital has therefore made significant progress in terms of volume.

Although the Blue Distinction criteria for joint replacement centers were created as quality standards for U.S. hospitals, using these criteria as a benchmark for evaluating TJR medical mission trips can help demonstrate care quality and identify areas of quality improvement. Since some aspects of the Blue Distinction criteria require organizations to report to US-based quality improvement organizations, future work should alter the existing criteria so that these organizations can earn points for reporting to equivalent international quality improvement organizations or provide waivers for organizations that operate in countries without equivalent quality improvement organizations. Furthermore, some Blue Distinction criteria require investment in patient navigators or expensive health care infrastructure, which is difficult because cost is a common barrier for most international medical missions. The criteria should therefore be redesigned so that they can be implemented with a level of investment more congruent with the resource capacity in the country being evaluated. Having a revised set of criteria could help medical mission trips better evaluate their own programs and allow them to enhance the care they provide. In the meantime, other international TJR mission programs might consider using the Blue Distinction criteria—or other similar criteria—to evaluate and improve their own programs, as our study demonstrates that these analyses can facilitate programmatic improvement.

### Limitations

This study’s data were potentially subject to observer bias, as data were collected by an investigator rather than a research assistant blinded to the study’s hypotheses and objectives. Anticipating this bias, we used data elements from the Blue Cross/Shield’s excellence criteria that were objective, binary, and subject to little interpretation. Furthermore, the data for this study was collected from a small number of respondents who provided key information to evaluate if the program meets or does not meet the criteria; it is therefore possible that responses would have been more heterogeneous if more people were surveyed but this variability should be limited by the objective and binary nature of the Blue Cross/Shield’s criteria.

## Conclusion

Op-Walk Boston qualified for Blue Distinction. Our analysis highlights areas of programmatic improvement and identifies targets for future quality improvement initiatives. Additionally, we note that many criteria can only be met by hospitals operating in the U.S. Future work should therefore focus on creating criteria that are applicable to TJR mission trips in the context of developing countries.

## Ethical approval

This study was approved by the IRBs at both the Brigham and Women’s Hospital in Boston and the Hospital General de la Plaza de la Salud in Santo Domingo.

## Competing interests

The authors of this manuscript report no relevant financial or other competing interests.

## Authors’ contributions

Conception: KED; JNK, Design: KED; RG; DD; LA; CB; BA; MH; TST; JNK, Data collection: KED, Data analysis: KED; JNK, Manuscript draft & revision: KED; RG; DD; LA; CB; BA; MH; TST; JNK. All authors have approved the final version of this manuscript in its entirety.

## Pre-publication history

The pre-publication history for this paper can be accessed here:

http://www.biomedcentral.com/1471-2474/14/275/prepub
